# Terahertz Spoof Surface Plasmon Polariton Waveguides: A Comprehensive Model with Experimental Verification

**DOI:** 10.1038/s41598-019-44029-1

**Published:** 2019-05-20

**Authors:** Muhammed Abdullah Unutmaz, Mehmet Unlu

**Affiliations:** 1 Ankara Yildirim Beyazit University, Ankara, 06010 Turkey; 2 TOBB University of Economics and Technology, Ankara, 06560 Turkey

**Keywords:** Terahertz optics, Nanophotonics and plasmonics, Characterization and analytical techniques, Sub-wavelength optics

## Abstract

Spoof surface plasmon polariton waveguides are perfect candidates to enable novel, miniaturized terahertz integrated systems, which will expedite the next-generation ultra-wideband communications, high-resolution imaging and spectroscopy applications. In this paper, we introduce, for the first time, a model for the effective dielectric constant, which is the most fundamental design parameter, of the terahertz spoof surface plasmon polariton waveguides. To verify the proposed model, we design, fabricate and measure several waveguides with different physical parameters for 0.25 to 0.3 THz band. The measurement results show very good agreement with the simulations, having an average and a maximum error of 2.6% and 8.8%, respectively, achieving 10-to-30 times better accuracy than the previous approaches presented in the literature. To the best of our knowledge, this is the first-time investigation of the effective dielectric constant of the terahertz spoof surface plasmon polariton waveguides, enabling accurate design of any passive component for the terahertz band.

## Introduction

Surface plasmon polaritons (SPP) are surface waves that propagate at the interface between dielectrics and metals at the optical frequency band, where the metals show complex and frequency dependent material characteristics, rather than behaving similar to perfect-electric-conductors (PEC)^1^. Similarly, surface waves propagating through the engineered metal surfaces at lower frequency bands such as millimetre-wave and terahertz (THz) bands are shown to mimic SPP waves, where the metals do not exhibit dispersive dielectric material properties^2–6^. These surface waves, which are often referred as spoof surface plasmon polaritons (sSPP), attracted interest of the researchers for the last decade having a potential for the terahertz high-speed communication^7–9^, imaging^10,11^ and sensing^12^ applications due to the high subwavelength confinement at the metal-dielectric boundary^3–6^. Several groups proposed printed circuit board-based proof of concept designs and sensing applications. These studies include TEM-mode to sSPP-mode transitions^13^, investigation of the sSPP WGs and sSPP filters^14–20^, metasurfaces^21^, couplers^22,23^, power dividers^24^, antennas^25–28^, switches^29^, mode splitters^30^ and sensors^31–33^. In all these studies, the main building block is the sSPP WG, and the physical parameters of the WG directly affect the performance of the proposed structure. On the other hand, none of these studies have employed a direct model for the effective dielectric constant of the sSPP WGs, which is the fundamental parameter determining the electrical length, and used indirect approximate solutions. Kianinejad *et al*. proposed a lumped-element model for a single-side corrugated sSPP WG and an sSPP-based meander transmission line is proposed at the microwave band^34,35^. The proposed model uses a single set of physical dimensions, and no effective dielectric constant model is used for the sSPP WG; instead, it is extracted using a finite element method (FEM) solver. Zhao *et al*. suggested modelling the corrugations of the single-side corrugated sSPP WG by periodically placed stubs on a single conductor transmission line. Here, the authors assumed no coupling between the stubs that are very closely spaced by a subwavelength separation^36^, which decreases the accuracy of the approach. Liu *et al*. considered the corrugations of a single-side corrugated sSPP WG as coplanar striplines (CPS) whose fundamental propagation mode is TEM^37^. This is in contrast with the fact that the sSPP WG supports TM mode surface waves. In addition, the corrugation depth and substrate thickness are not taken into the account, both of which reduce the accuracy of the approximation. An effective dielectric constant model for the sSPP WGs is introduced for the microwave band^38^. This solution is examined only at a single frequency, 6 GHz, for a limited set of physical parameters; moreover, the corrugation depth, which is one of the most fundamental parameters, is kept constant for all the measurements. Above all, the dielectric thicknesses in this study, as well as all above-mentioned studies, are significantly lower than the free-space wavelength at their corresponding operation frequencies, which address a totally different problem compared to proposed model. Although few sSPP waveguides are also presented for the terahertz integrated circuits^39,40^, they also follow a similar approximation to that of the studies presented in the microwave bands, and neither an effective dielectric constant model, nor its experimental verification is presented so far for the terahertz band. In particular, the effective dielectric constants in these studies are extracted from a limited set of physical dimensions and no model that governs the effects of the variations in all the physical parameters is presented. Hence, an accurate universal model for the effective dielectric constant that takes the variations in the physical parameters and operation frequency into consideration is needed for the terahertz band.

In this paper, we present an empirical model for the effective dielectric constant of the double-side corrugated sSPP WG for the terahertz band. The proposed model is not only the first model presented in the literature for the terahertz band, but also comprises the effects of all the physical parameters of the sSPP WG, namely corrugation depth, aperture width, periodicity, permittivity of the substrate, metal thickness and substrate thickness. We also verify the performance of the proposed model with the measurement results of several sSPP WGs at the terahertz band, which are also presented for the first time and show very good agreement with the simulations. According to the measurement results, the model shows at least an order of magnitude better accuracy at 0.25 to 0.3 THz band, compared to the previous indirect approximate solutions. Consequently, our model enables much more accurate design of the sSPP WGs, which is one of the most critical components for high performance integrated systems for terahertz imaging, sensing and communication applications.

## Results

**Figure 1:**
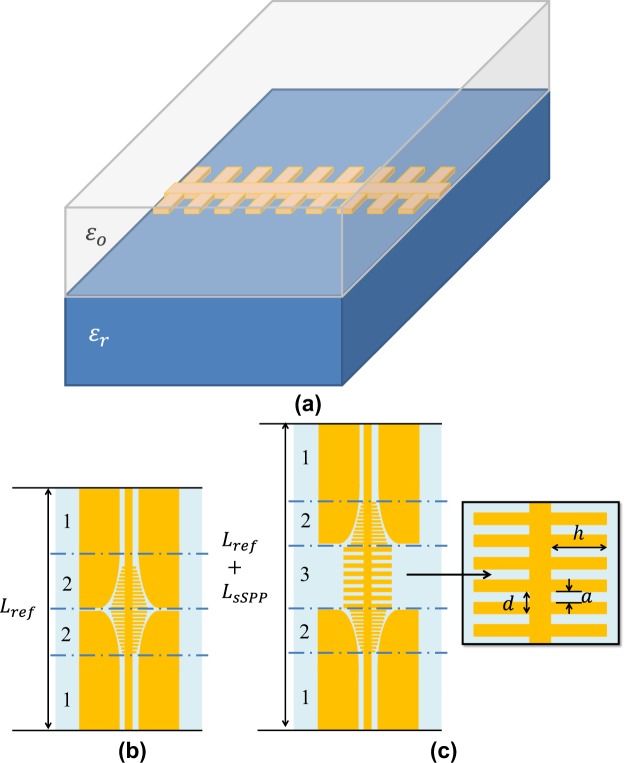
The schematic of the corrugated planar sSPP WG (**a**). The layout of the reference line (**b**) and the sSPP delay line with cell dimensions (**c**). In the waveguides, (1) corresponds to the coplanar waveguide sections, (2) to the transition sections and (3) to the sSPP delay sections (3). The physical dimensions of the sSPP WGs (corrugation depth (*h*), periodicity (*d*) and aperture width (*a*)) are also shown.

The schematic of the sSPP WG is presented in Fig. 1(a). The WG is composed of a planar, double-side corrugated, single conductor, which is placed on the surface in between air and a dielectric substrate. The WG can be considered as the cascade connection of the single periods of the sSPP cells. The effective dielectric constant is extracted using Eq. (1) by comparing the electrical lengths of the delay lines (DLs) with a reference line as depicted in Fig. 1(b).

1 $${\varepsilon }_{eff}={({\rm{\Delta }}\rlap{/}{0}/{k}_{o}{L}_{sSPP})}^{2}$$

*ε*_*eff*_ is the effective dielectric constant, ${\rm{\Delta }}\rlap{/}{0}$ is the phase difference between the DL and reference line. *k*_*o*_ is the free-space wavenumber at the frequency of operation and ${L}_{sSPP}$ is the physical length difference between DLs and the reference line. The sSPP delay section is available only in DLs, where the delay section is only composed of the sSPP cells. Every sSPP cell has six different WG parameters, namely corrugation depth (*h*), periodicity (*d*), aperture width (*a*), metal thickness (*t*_*met*_), substrate permittivity (*ε*_*r*_) and substrate thickness (*t*_*sub*_).

The comparison of the electrical lengths of the DLs and reference line is obtained by finite element method (FEM) simulations, which are performed using a commercial solver, ANSYS HFSS. In order to excite the sSPP waves for the simulations and measurements, coplanar waveguides (CPW) and CPW-to-sSPP WG transitions are used, which can also be observed in Fig. 1(c). The transition section is first designed without having sSPP delay sections to maximise the momentum matching between TEM CPW and TM sSPP modes in a similar way to Ma *et al*.^13^. Here, the corrugation depth (*h*) in the transition region has been tapered and the CPW ground has been flared with an elliptical path so that the return loss, insertion loss and radiation loss of the transition section has been minimised. The usage of CPW and CPW-to-sSPP transitions also guarantees that the simulation and measurement environment have the same physical conditions. The dimensions of CPW and transition sections are given in the Supplementary Information Table S1.

The effect of each parameter on the effective dielectric constant has been investigated separately by adjusting only one parameter at a time. The metals have first been assumed as perfect-electric-conductors (PEC) for the effective dielectric constant extraction and then the simulations are repeated including the metal properties. The set of the simulation scenarios are given in the Supplementary Information Table S2, where a wide set of dimensions have been investigated in order to observe delay characteristics of the sSPP WGs. Set 1 and Set 2 are dedicated to get insight about the effects of corrugation depth, *h*, on the insertion phase. Then, the effect of the aperture width, *a*, is investigated for the cases given in Set 3 to Set 6. The substrate thickness is also investigated in Set 7, where the substrate thickness, *t*_*sub*_, is changed to the typical thickness of a 4” silicon wafer, rather than the typical thickness of a 6” silicon wafer, as used in Set 1-to-6. Here, the effect of the substrate thickness is chosen considering the largest radial decay for the sSPP waves. Set 8 is used to observe the effects of relative permittivity, *ε*_*r*_.

The idea behind the formulation of the effective dielectric constant is to represent the effects of the corrugations and two dielectric layers on both sides of the corrugated metal layer on the electrical length of the waveguide. For this purpose, a conformal mapping technique is employed that maps a two-dimensional, corrugated half-space to a smooth-boundary half-space^41^. Here, the conformal mapping function in ref.^41^ is the main part of Eq. (2), i.e., the fourth summation term composed of the subfunctions *F*_1_, *F*_2_, *F*_3_, *F*_4_ and *F*_5_ (Eqs (3) to (7)). The remaining terms in Eq. (2) is used for the empirical modelling of the effects of the finite metal thickness of the corrugated surface and two layers of dielectric on both sides of the corrugated metal. The Eqs (8) to (10) represent the parameters used in Eqs (4) and (5), and Eqs (11) to (14) are the subsequent parameters and functions used in Eq. (10). Here, $F(.,.)$ and $F(.\,)$ are the elliptic functions of the first kind, *SN*_*i*_ are the elliptic sinuses and ${\prod }^{}(.,.,.)$ is the elliptic integral of the third kind^41,42^.

2 $${\varepsilon }_{eff}=\{\begin{array}{cc}1+\frac{0.5-2500{t}_{met}}{3{\lambda }_{o}}+{(\frac{6{\varepsilon }_{r}}{11.9})}^{3}+1.25\times {10}^{-4}{f}_{GHz}{e}^{3{h}_{n}}(50-22.5\sqrt{{F}_{1}}{F}_{2}({F}_{3}{F}_{4}+{F}_{5})-{e}^{\frac{1}{{d}_{n}}})+1.5\times {10}^{-3}{e}^{3{h}_{n}}{f}_{GHz}+\sqrt{\frac{0.005{h}_{n}}{{d}_{n}}} & if\,{\varepsilon }_{r}\ge 11.9\\ 1+\frac{0.5-2500{t}_{met}}{3{\lambda }_{o}}+{(\frac{6{\varepsilon }_{r}}{11.9})}^{3}+7.5\times {10}^{-5}{f}_{GHz}{e}^{3{h}_{n}}(50-22.5\sqrt{{F}_{1}}{F}_{2}({F}_{3}{F}_{4}+{F}_{5})-{e}^{1/{d}_{n}})+3\times {10}^{-3}{e}^{3{h}_{n}}{f}_{GHz}+\sqrt{\frac{0.005{h}_{n}}{{d}_{n}}} & otherwise\end{array}$$

3 $${F}_{1}=-\,2({\varepsilon }_{r}+1)$$

4 $${F}_{2}=\frac{2{d}_{n}}{\pi \sqrt{(1+{d}_{n}-{a}_{n})(1+{h}_{n})}}$$

5 $${F}_{3}={d}_{n}-{a}_{n}-1$$

6 $${F}_{4}=F(m\,\tanh (0.5w),k)$$

7 $${F}_{5}=2\prod (2\,\tan (0.5w),{m}^{2},k)$$

8 $$m=\sqrt{\frac{1-{h}_{n}}{1+{h}_{n}}}$$

9 $$k=\sqrt{\frac{(1-{d}_{n}+{a}_{n})(1+{h}_{n})}{(1+{d}_{n}-{a}_{n})(1-{h}_{n})}}$$

10 $$w=\frac{{d}_{n}}{{K}_{n}}F(S{N}_{1}/S{N}_{2})$$

11 $$S{N}_{1}=\,\tanh (\frac{{K}_{n}{h}_{n}}{{d}_{n}})-\frac{0.25{k}^{\text{'}2}(\frac{{K}_{n}{h}_{n}}{{d}_{n}}-\,\sinh (\frac{{K}_{n}{h}_{n}}{{d}_{n}})\cosh (\frac{{K}_{n}{h}_{n}}{{d}_{n}}))}{4{\cosh }^{2}(\frac{{K}_{n}{h}_{n}}{{d}_{n}})}\,$$

12 $$S{N}_{2}=\,\tanh (i{h}_{n}+0.5)-\frac{0.25{k}^{\text{'}2}(i{h}_{n}+0.5-\,\sinh (i{h}_{n}+0.5)\cosh (i{h}_{n}+0.5))}{4{\cosh }^{2}(i{h}_{n}+0.5)}\,$$

13 $$k^{\prime} =\sqrt{1-{k}^{2}}$$

14 $${K}_{n}=F(\pi /2,k)$$

In the formulation, ${f}_{GHz},\,{\lambda }_{o},\,{h}_{n},\,{a}_{n}$ and *d*_*n*_ are frequency of operation in GHz, free-space wavelength at the frequency of operation and normalised physical dimensions of the sSPP WG, respectively. A mapping wavelength is used for the normalization of the physical dimensions, which is empirically determined at the corresponding mapping frequency, where the complex analysis solution^41^ requires detailed region-of-convergence analysis. The mapping frequencies used for the simulations and measurements are given in the Supplementary Information Table S3.

**Figure 2:**
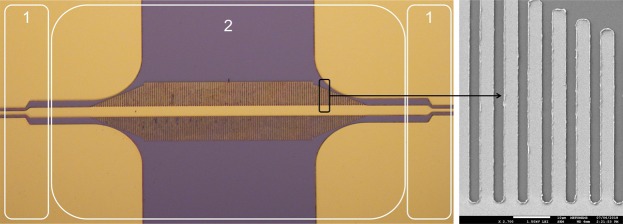
The microscope and SEM images of one of the fabricated sSPP WG (Set 1, h = 57.5 μm). CPW connections between the contactless probes and original devices (1) and original device (2).

**Figure 3:**
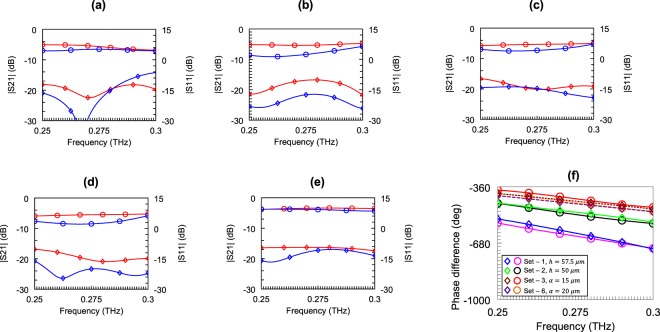
The comparison of the measured and simulated S-parameters (measurements (S_11_, S_21_ (, )) and simulations ((, ))): Set 1, *h* = 57.5 μm (**a**), Set 2, *h* = 50 μm (**b**), Set 3, *a* = 15 μm (**c**), Set 6, *a* = 20 μm (**d**), and reference line (**e**). Relative insertion phases of the delay lines with respect to the reference line: Set 1, *h* = 57.5 μm, Set 2, *h* = 50 μm, Set 3, *a* = 15 μm and Set 6, *a* = 20 μm, measurements (, ○, , ) and simulations (, , , ), respectively (**f**).

A subset of the simulation sets is opted for fabrication to verify the performance of the proposed formula. The list of the fabricated devices is given in Table 1. High-resistivity (>10 kΩ-cm), 4″ silicon wafer with a permittivity of 11.65^43^ is used as the substrate and the metal thickness is selected as 540 nm Au. The microscope and SEM images of one of the fabricated devices are given in Fig. 2. The S-parameters of the fabricated sSPP WGs are measured in 0.22 to 0.32 THz band using a contactless probe measurement method^44^. The measurement results of devices from different simulation sets show good agreement with the simulations, which are presented in Fig. 3. The difference between the simulated and measured S_21_ magnitudes are caused by the ohmic losses due to the high-resistivity Cr layer that is used as the adhesion layer between the Au layer and silicon substrate. Then, the effective dielectric constant is extracted from the measurements using the same method that had been applied for the simulations. The measured effective dielectric constants which were obtained from 21 different sSPP WGs show very good agreement with the proposed model, having an average and a maximum error of 2.6% and 8.8%, respectively. The error rates between the measurements and model for a wide range of parameters, each of which influences the effective dielectric constant, are presented in Table 1. The mapping frequencies used for the formulation for each case are presented in Table S3. The slight variation between the mapping frequencies used for the measurements and model emerges due to the systematic variation of the physical dimensions of the fabricated devices caused by the optical lithography tolerances. It is also observed that the difference in the S_21_ amplitude levels has no noticeable effect on the performance of the proposed model.

**Table 1:** The list of the measured WGs and corresponding error rates, model (Model) vs. measurements (Meas.).

Set	Adjusted Parameter (μm)	0.25 THz			0.275 THz			0.3 THz		
		Meas.	Model	Error (%)	Meas.	Model	Error (%)	Meas.	Model	Error (%)
1	*h* = 32.5	12.2	12.3	0.8	13.1	13.0	0.8	14.7	13.5	8.2
1	*h* = 35	13.2	12.9	2.3	13.9	13.6	2.2	14.2	14.2	0.0
1	*h* = 42.5	15.4	15.0	2.6	16.2	16.0	1.2	16.5	16.8	1.8
1	*h* = 47.5	17.3	16.8	2.9	18.2	18.0	1.1	18.5	18.9	2.2
1	*h* = 57.5	22.1	21.5	2.7	23.4	23.3	0.4	24.1	24.7	2.5
1	*h* = 70	31.0	29.6	4.5	33.0	33.4	1.2	36.8	37.7	2.4
1	*h* = 75	34.8	34.3	1.4	37.7	39.0	3.4	43.1	44.4	3.0
2	*h* = 25	9.1	9.9	8.8	9.6	10.3	7.3	10.1	10.7	5.9
2	*h* = 30	10.1	10.4	3.0	10.4	10.9	4.8	10.8	11.3	4.6
2	*h* = 40	11.5	11.6	0.9	12.3	12.4	0.8	12.6	13.1	4.0
2	*h* = 50	13.9	13.3	4.3	14.9	14.3	4.0	15.1	15.4	2.0
2	*h* = 70	22.2	22.0	0.9	23.7	23.5	0.8	25.3	25.9	2.4
2	*h* = 75	24.4	24.7	1.2	27.3	26.5	2.9	30.6	29.4	3.9
3	*a* = 10	10.5	10.6	1.0	11.1	11.1	0.0	11.5	11.6	0.9
3	*a* = 15	10.7	10.6	0.9	11.2	11.1	0.9	11.7	11.6	0.9
4	*a* = 10	9.8	10.1	3.1	10.2	10.5	2.9	10.6	10.8	1.9
4	*a* = 25	10.4	10.1	2.9	10.7	10.5	1.9	11.1	10.8	2.7
5	*a* = 5	7.8	8.3	6.4	8.1	8.5	4.9	8.6	8.9	3.5
5	*a* = 35	8.5	8.3	2.4	8.7	8.5	2.3	9.2	8.9	3.3
6	*a* = 20	9.6	9.4	2.1	10.2	10	2.0	10.6	10.4	1.9
6	*a* = 40	9.3	9.4	1.1	9.8	10	2.0	10.2	10.4	2.0

**Figure 4:**
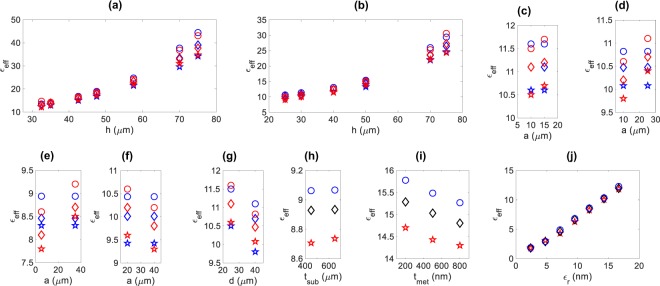
The comparison of measured and modelled effective dielectric constants: variation with respect to *h* (Set 1 and 2) (**a,b**), variation with respect to *a* (Set 3 to 6) (**c**–**f**) and variation with respect to *d* (*a* = 10 μm and *h* = 30 μm) (**g**). The symbols in the figures correspond to the model at 0.25 THz (), 0.275 THz () and 0.3 THz (), and measurements at 0.25 THz (), 0.275 THz () and 0.3 THz (). The simulated variation of the effective dielectric constant with respect to substrate thicknesses (Set 7) (**h**), metal thickness (Set 9) (**i**) and relative permittivity (Set 8) **(j**). The symbols in the figures of simulations correspond to 0.25 THz (), 0.275 THz (◊) and 0.3 THz ().

The measured and modelled effective dielectric constants with respect to the corrugation depth, *h*, aperture width, *a*, and periodicity, *d*, are given in Fig. 4, which confirms the reliability of the proposed model. The effective dielectric constants of the sSPP WGs with *h*, *a* and *d* in the ranges of 25 to 75, 20 to 40 and 25 to 40 μm vary from 9 to 43, 8.5 to 11.5 and 9.8 to 11.5 in the frequency region of interest, respectively. The corrugation depth, *h*, is the most dominant parameter and results in the largest change in the effective dielectric constant. Increasing the periodicity, *d*, with a constant aperture width, *a*, decreases the effective dielectric constant; while, increasing *a* with a constant *d* provides an increase in the effective dielectric constant as expected.

Figure 4 shows dependence of the effective dielectric constant with respect to the substrate thickness, metal thickness and relative permittivity that are examined using the simulations. The effective dielectric constants show an imperceptible variation with the substrate thickness, even in the case of largest radial decay. Contrarily, the metal thickness affects the effective dielectric constant to some extent, where a 300 nm change in the metal thickness causes about 4% change. The relative permittivity of the substrate has a direct impact on the effective dielectric constant as expected. All these results verify the expected behaviour of the respective changes in the examined physical parameters.

Considering the range of all the parameters examined, the model can be applied to any range of physical parameters, provided that the ratio of the physical parameter with respect to the operating wavelength remains the same. However, it is also observed that the error of the proposed model starts to increase when the corrugation depth, (*h*), becomes very small, which is due to the decreasing confinement of the sSPP waves, and very large, which is due to the operation in the cut-off region. In addition, the thickness of the substrate (*t*_*sub*_) should not be very small compared to the operating wavelength.

**Figure 5:**
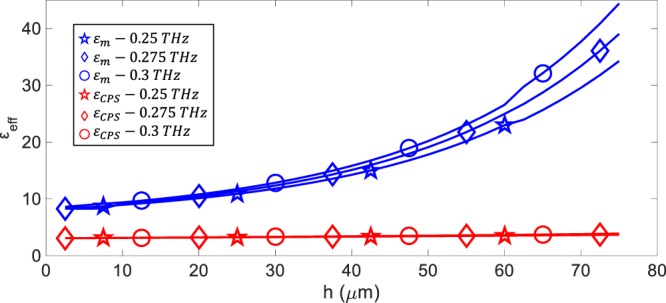
The comparison of the proposed effective dielectric constant model (*ε*_*m*_ at 0.25 THz, 0.275 THz and 0.3 THz) with the CPS approximation (*ε*_*CPS*_ at 0.25 THz, 0.275 THz and 0.3 THz): proposed model (*h* = 2.5-to-75 μm, *d* = 5 μm, *a* = 2.5 μm, *t*_*met*_ = 540 nm and *ε*_*r*_ = 11.65). Here, *ε*_*CPS*_^37^ is calculated using ${\varepsilon }_{CPS}={({k}_{x}/{k}_{0})}^{2}$.

The performance of the proposed model is also compared with that of the only alternative method presented in the literature^37^. In the alternative method, the corrugations of the sSPP WG are approximated using coplanar striplines (CPS). This CPS approximation is applied to all the fabricated sSPP WGs, and the calculations show that the CPS approximation results in effective dielectric constants ranging from 3 to 4, with a best-case error of 74.9% and an average error rate of 84.0%. On the other hand, the average and worst-case errors of the proposed model are 2.6% and 8.8%, respectively. Figure 5 shows an example case for the comparison for the variation of the corrugation depth, *h*, for the set 1. These results clearly show that the proposed model provides a substantial improvement over the CPS approximation, decreasing the error rates by 10 to 30 times. Hence, the proposed model offers a very promising solution for the terahertz band and allows the accurate design of any passive circuit that are based on the promising sSPP WGs. Furthermore, the proposed model utilizes unitless dimensions, so that it can be used at higher frequency bands, such as infrared and optical wavelengths, once the dimensions and mapping wavelengths are scaled for the wavelengths of interest.

## Discussion

We present a model for the most critical parameter, namely, the effective dielectric constant, of a spoof surface plasmon polariton waveguide for the terahertz band. The reliability of the model is justified by the fabrication and measurements for 0.25 to 0.3 THz band. Not only the simulations and measurements agree well with each other, but also the formula meets an average error of 2.6% and a worst case error of 8.8%, providing a very significant improvement of at least an order of magnitude over the only alternative solution^37^. The proposed formula is based on detailed analysis of all of six parameters of the sSPP WG, which are material thicknesses and corrugation dimensions.

The sSPP WGs can provide a high-performance alternative for the next generation THz integrated systems, where the propagation of the THz signals can be confined to much lower physical dimensions, increasing the scalability and speed of the THz integrated systems, while reducing the cross-talk and signal integrity problems. The proposed model is critical in the sense that it directly determines the electrical length, hence, the performance of any given passive component based on sSPP WGs, such as waveguides, couplers, power dividers, filters, resonators and antennas, which are all fundamental parts of a THz integrated system. The performances of these components are all crucial for the system level performance and the improvement of the performances of these components will improve the scarce THz system performance significantly.

With the employment of the proposed model, the sSPP WGs become perfect alternatives for the realization of the miniaturized THz integrated systems for ultra-wideband communications, high-resolution imaging and spectroscopy applications. Additionally, this model can also be utilized for higher frequency bands, provided that the dimensions are normalised with respect to the frequency of interest.

## Methods

### Simulations

For the simulations of the proposed sSPP WGs, a commercial FEM solver, ANSYS HFSS, is utilized. Here, the excitations are selected as the wave ports and boundaries are set as radiation boundaries. The solution frequency is set to 0.35 THz, and the frequency sweep is selected from 0.001 to 0.35 THz with a step size of 10^−4^ THz.

### Fabrication

sSPP WGs are fabricated at the METU-MEMS Research and Application Centre, Ankara, Turkey. The metal layer is coated by sputtering 20/540 nm Cr/Au and the lift-off method is used for patterning the metal layer. The substrate is a 4″ Si substrate with a resistivity of 10 kΩ-cm and thickness of 425 μm. The process flow and microscope image of a fabricated device is given in the Supplementary Information Fig. S1.

### Measurements

Contactless probing solution provided by TeraProbes, Inc. is used for the S-parameter measurements. On-wafer Quick Offset Shorts (Q-SSS) method is used for the calibration^44^. The calibration is verified by comparing the simulation result of some known structures before the sSPP WGs are measured.

## Supplementary information

Supplementary information
