# Supplementary material for: Terahertz Spoof Surface Plasmon Polariton Waveguides: A Comprehensive Model with Experimental Verification
Source: Sci Rep. 2019 May 20;9:7616. doi: 10.1038/s41598-019-44029-1 (PMC6527547; doi:10.1038/s41598-019-44029-1)
Supplement: Supplementary file 1 — Supplementary information [file 41598_2019_44029_MOESM1_ESM.pdf]

Supplementary Information for “Terahertz Spoof Surface Plasmon Polariton Waveguides: A Comprehensive Model with Experimental Verification”

[1]Muhammed Abdullah Unutmaz and [2]Mehmet Unlu*

[1] Ankara Yildirim Beyazit University, Ankara, Turkey, 06010

[2] TOBB University of Economics and Technology, Ankara Turkey, 06560

E-mail addresses: maunutmaz@ybu.edu.tr and (2) munlu@etu.edu.tr

Table S1| The dimensions of CPW and transition sections.	
CPW length	253 µm
CPW gap	15 µm
CPW pitch	55 µm
Period length in transition	5 µm
Aperture length in transition	2.5 µm
Maximum corrugation depth in transition	50 µm
Minimum corrugation depth in transition	2 µm
Transition section length	125 µm
Major radius in transition	100 µm
Major to minor Radius rate in transition	1.2

Table S2| The dimensions and physical properties of the sSPP WG simulation sets.

Set #	Delay Section Length (µm)	a (µm)	d (µm)	h (µm)	t_{sub} (µm)	$^*t_{met}$ (nm)	ε_r	Step Size
1	402.5	2.5	5	2.5 to 75	650	100 nm	11.9	2.5 µm
2	407.5	5	25	5 to 75	650	100 nm	11.9	5 µm
3	407.5	5 to 20	25	30	650	100 nm	11.9	5 µm
4	407.5	5 to 30	40	30	650	100 nm	11.9	5 µm
5	407.5	5 to 45	50	20	650	100 nm	11.9	5 µm
6	407.5	5 to 45	50	30	650	100 nm	11.9	5 µm
7	402.5	2.5	5	2.5	450	100 nm	11.9	-
8	402.5	2.5	5	2.5	650	100 nm	2.38 to 16.66	2.38
9	402.5	2.5	5	35	650	200 nm to 800 nm	11.9	300 nm

*Set 1-to-Set 8 metals are of PEC type and gold (Au) is used in Set 9.

Table S3| The mapping frequencies for the simulations (f_{ms}) and measurements (f_{mm}).

Set #	Adjusted Parameter	Range (µm)	$f = 0.25\,THz$		$f = 0.275\,THz$		$f = 0.3\,THz$	
			f_{ms} (THz)	f_{mm}/f_{ms}	f_{ms} (THz)	f_{mm}/f_{ms}	f_{ms} (THz)	f_{mm}/f_{ms}
1	h	[2.5, 10]	$900/f_{GHz}$	-	$1000/f_{GHz}$	-	$1180/f_{GHz}$	-
1	h	[10, 62.5]	$925/f_{GHz}$	1.0270	$1050/f_{GHz}$	1.0048	$1180/f_{GHz}$	0.9746
1	h	(62.5,75]	$900/f_{GHz}$	1.0389	$1062.5/f_{GHz}$	0.9906	$1205/f_{GHz}$	0.9751
2	h	[2.5, 65)	$700/f_{GHz}$	1.0000	$800/f_{GHz}$	1.0000	$900/f_{GHz}$	1.0000
2	h	[65, 75]	$775/f_{GHz}$	1.0258	$850/f_{GHz}$	1.0294	$975/f_{GHz}$	1.0000
3	a	Entire set	$700/f_{GHz}$	1.0714	$750/f_{GHz}$	1.1333	$750/f_{GHz}$	1.2667
4	a	Entire set	$600/f_{GHz}$	1.0667	$670/f_{GHz}$	1.0746	$650/f_{GHz}$	1.2154
5	a	Entire set	$150/f_{GHz}$	0.8000	$200/f_{GHz}$	0.7000	$225/f_{GHz}$	1.6000
6	a	Entire set	$550/f_{GHz}$	0.8636	$610/f_{GHz}$	1.0000	$600/f_{GHz}$	1.1667

f_{GHz} is defined as the operation frequency in GHz.

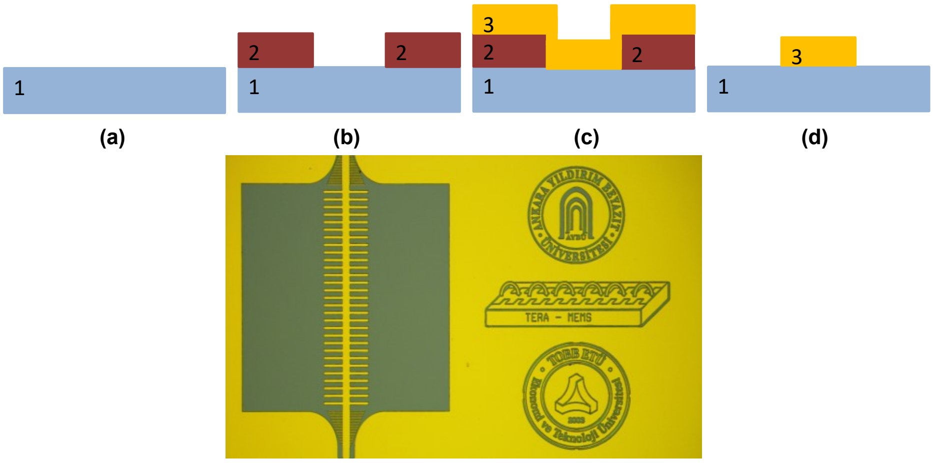

(e)

Figure S1| Fabrication steps: High-resistivity silicon wafer (a), lithography using SPR220-3 photoresist (b), Cr (20 nm)/Au (540 nm) sputtering (c), lift-off (d) and microscope image of a fabricated waveguide (e).
